# Ecologic and Geographic Distribution of Filovirus Disease

**DOI:** 10.3201/eid1001.030125

**Published:** 2004-01

**Authors:** A. Townsend Peterson, John T. Bauer, James N. Mills

**Affiliations:** * University of Kansas, Lawrence, Kansas, USA; † Centers for Disease Control and Prevention, Atlanta, Georgia, USA

**Keywords:** filoviridae, filovirus, hemorrhagic fever, Ebola virus, Marburg virus, mathematical model, ecologic niche

## Abstract

We used ecologic niche modeling of outbreaks and sporadic cases of filovirus-associated hemorrhagic fever (HF) to provide a large-scale perspective on the geographic and ecologic distributions of Ebola and Marburg viruses. We predicted that filovirus would occur across the Afrotropics: Ebola HF in the humid rain forests of central and western Africa, and Marburg HF in the drier and more open areas of central and eastern Africa. Most of the predicted geographic extent of Ebola HF has been observed; Marburg HF has the potential to occur farther south and east. Ecologic conditions appropriate for Ebola HF are also present in Southeast Asia and the Philippines, where Ebola Reston is hypothesized to be distributed. This first large-scale ecologic analysis provides a framework for a more informed search for taxa that could constitute the natural reservoir for this virus family.

The natural maintenance cycles of filoviruses (Order *Mononegavirales*, family *Filoviridae*) are unknown *(1)*.^1^Although dynamics of filoviruses as causes of epidemic diseases among humans, great apes, and other primates have been described in detail (*2*–*13*), the natural reservoir, mode of transmission to hominids and pongids (humans, gorillas, and chimpanzees), and temporal dynamics remain unclear. Diverse taxa have been suggested as ^potential^ reservoirs, including bats, rodents, arthropods, and plants (*14*–*18*).

 Two observations provide clues about the nature of the host-virus relationship. First, filovirus transmission to humans is not common, and most occurrences can be traced to a single index case (*2*,*6*,*19*) (exceptions occur—e.g., the Durba Marburg outbreak appears to have involved multiple independent infections of humans from a reservoir population presumably associated with a mine). We assume that introductions to nonhuman hominid populations also generally begin with single index cases, but this hypothesis is more difficult to investigate. This rarity argues against a common arthropod vector for transmission: if anthrophilic arthropod vectors were to carry filoviruses, multiple index cases would be more common, as many hominids in an area would have the opportunity for infection. In addition, filoviruses generally do not replicate in arthropods or arthropod cell lines, leading several authors to speculate on more incidental modes of transfer (e.g., direct contact) (*20*).

 Second, filoviruses show clear geographically related phylogeographic structure. Viruses and subtypes from particular geographic areas cluster together phylogenetically, even when occurrences from different years are studied. This phylogeographic structure suggests a stable host-parasite relationship, in which viruses are maintained in permanent local-regional pools. This host would not experience high death rates, as primates do (*7*,*9*); evolution of avirulence in long-term host-parasite relationships is expected on theoretical grounds (*21*).

 Searches for the natural reservoir of filoviruses have taken several paths. Epidemiologic studies designed to trace lineages of transmission in outbreaks have identified index cases but have not succeeded in specifying the mode of “jump” to hominids (*2*,*6*,*19*). Testing large numbers of organisms from the vicinity of outbreaks has failed to identify even a single nonhominid infection (*14*–*16*). Finally, laboratory tests of reservoir competence of species have documented the following: 1) no, or very limited, infection of plants or arthropods; 2) a single marginally successful infection of snakes but with very low levels of virus circulation; 3) successful infection of bats and possibly rodents; and 4) frequent successful, but fatal, infection in nonhuman primates (e.g., *Chlorocebus*, *Macaca*) (*1*,*17*,*18*). While these investigations have shed some light, they have not provided convincing evidence for a particular reservoir.

An unexplored approach to identifying the natural reservoir of filoviruses is large-scale ecologic and geographic comparisons to detect patterns of co-occurrence and codistribution of viruses with potential hosts. This approach has been applied successfully to identifying reservoir rodent species for Chagas disease (*22*). Our general approach is as follows: 1) to understand the large-scale ecology and geography of disease occurrences by using ecologic niche modeling (*23*), and 2) to compare these characteristics with ecologic and geographic patterns of potential reservoirs. Here, we address the first step and document broad-scale ecologic and geographic patterns in filovirus-associated HF occurrences.

## Methods

Distributional data for filovirus-associated HF occurrences in hominids were accumulated from the literature (Table). Occurrences of unknown origin were excluded from analysis, but when reasonable guesses could be made as to point or general area of origin (e.g., 1995 outbreak of hemorrhagic fever due to Ebola Ivory Coast as originating at Plibo, Liberia), they were included. All occurrences were georeferenced (available from: URL: http://www.calle.com/world) to the nearest 0.001°. Although assigned geographic coordinates may not fix the exposure point precisely, they represent our best guess as to its position and are likely to be representative of the coarse-scale ecologic conditions. (The relatively crude spatial resolution at which analyses were conducted makes some error in georeferencing irrelevant.)

**Table:** Virus, location, dates, geographic coordinates, and literature citation for filovirus-caused hemorrhagic fever occurrences

Virus	Country	Apparent origin	Dates	Latitude	Longitude	Reference
Ebola Ivory Coast	Cote d’Ivoire	Tai National Park	Nov. 1994	5.850 5.900	-7.367 -7.317	(*7**,**24*)
Ebola Ivory Coast	Cote d’Ivoire or Liberia	Plibo (Liberia)	Dec. 1995	4.589	-7.673	(*25*)
Ebola Sudan	Sudan	Nzara	June–Nov 1976	4.643	28.253	(*3*)
Ebola Sudan	Sudan	Nzara	July–Oct 1979	4.643	28.253	(*4*)
Ebola Sudan	Uganda	Gulu	Oct 2000–Feb 2001	2.783	32.300	(*26*)
Ebola Zaire	DRC	Yambuku	Sept–Oct 1976	2.817	22.233	(*2*)
Ebola Zaire	DRC	Bonduni	June 1977	2.967	19.350	(*10*)
Ebola Zaire	Gabon	Minkebe,	Dec 1994–Feb 1995	1.733	12.817	(*8*)
		Mekouka,		1.400	12.983	
		and/or Andock		1.483	12.917	
Ebola Zaire	DRC	Kikwit	Jan–Jul 1995	-5.058	18.909	(*11*)
Ebola Zaire	Gabon	Mayibout	Feb 1996	-1.117	-13.100	(*8*)
Ebola Zaire	Gabon	Booue	Jul 1996–Mar 1997	-0.100	-11.95	(*8*)
Ebola Zaire	Gabon and DRC	Ekata	Dec 2001–2002	0.706	14.275	(*12*)
Marburg	Zimbabwe	Wankie?a	Feb 1975	-18.367	26.483	(*6*)
Marburg	Kenya	Nzoia or Mt. Elgon	Jan 1980	0.450	34.617	(*19*)
Marburg	Kenya	Mt. Elgon?	1987	1.133	34.550	(*20*)
Marburg	DRC	Durba	Apr 1999–Sept. 2000	3.117	29.583	(*27**–**29*)

^a^Reported location where patient received a “bite.” Although some investigators felt the disease was related to the bite, the patient had traveled widely in Zimbabwe and parts of South Africa and was exposed to wildlife at several locations in Zimbabwe (*6*). DRC, Democratic Republic of the Congo; WHO. World Health Organization.

Ecologic niches and potential geographic distributions were modeled by using the Genetic Algorithm for Rule-set Prediction (GARP) (*30*–*32*) (available from: URL: http://www.lifemapper.org/desktopgarp/). In general, GARP focuses on modeling ecologic niches (the conjunction of ecologic conditions wherein a species can maintain populations without immigration) (*33*). Specifically, GARP relates ecologic characteristics of occurrence points to those of points sampled randomly from the rest of the study region, developing a series of decision rules that best summarize factors associated with presence (*23*).

Occurrence points are divided evenly into training (for model building) and test (for model evaluation) datasets. GARP works in an iterative process of rule selection, evaluation, testing, and incorporation or rejection: a method is chosen from a set of possibilities (e.g., logistic regression, bioclimatic rules) and applied to the training data to develop or evolve a rule. Predictive accuracy is evaluated on the basis of the test data. Rules may evolve in ways that mimic DNA evolution (e.g., point mutations, deletions). Change in predictive accuracy between iterations is used to evaluate whether particular rules should be incorporated into the model; the algorithm runs 1,000 iterations or until convergence. Model quality was evaluated through independent test dataset reserved prior to modeling; a chi-square test was used to compare observed success in predicting the distribution of test points with that expected under a random model (proportional area predicted present provides an estimate of occurrence points correctly predicted, were the prediction random with respect to the distribution of the test points).

To characterize environments, we used 11 GIS coverages summarizing elevation, slope, aspect, flow direction, flow accumulation, and tendency to pool water (from the USGS Hydro-1K dataset [available from: URL: http://edcdaac.usgs.gov/gtopo30/hydro/), and climate characteristics, including daily temperature range; mean annual precipitation; maximum, minimum, and mean annual temperatures; solar radiation; frost days; wet days; and vapor pressure (1960–1990; Intergovernmental Panel on Climate Change [available from: URL: http://www.ipcc.ch/]). These coverages are worldwide and provide a consistent view of ecologic variation across regions studied. GARP’s predictive ability has been tested under diverse circumstances (*22*,*23*,*34*–*47*).

To optimize model performance, we developed 100 replicate models of ecologic niches based on independent random subsamples from available occurrences. We chose a “best subset” of these models on the basis of optimal error distributions for individual replicate models (*34*): median area predicted across all replicate modes was calculated, and the 20 models with predicted areas closest to the median were chosen for further consideration. These geographic predictions were combined to provide a summary of potential geographic distributions. Projection of the Africa-based rule-sets onto maps of Asia and the Pacific provided hypotheses of potential distributional areas in other regions (*46*).

To permit visualization of the ecologic dimensions of models, we combined best-subsets predictions with maps of the ecologic parameters used to build them in a GIS environment (COMBINE in ArcView 3.2). The resulting dataset represents unique combinations of environments and predictions; its attributes table provides the model prediction for all environmental combinations to permit visualization of ecologic variation. We also compared ecologic conditions inside and outside of the modeled Ebola HF distribution within 11 regularly spaced circular windows (radius 50 km); comparisons were summarized through Mann-Whitney U-statistics, permitting a nonparametric visualization of the strength of association of each ecologic dimension (temperature, precipitation, elevation) with the range limit.

## Results

**Figure 1:**
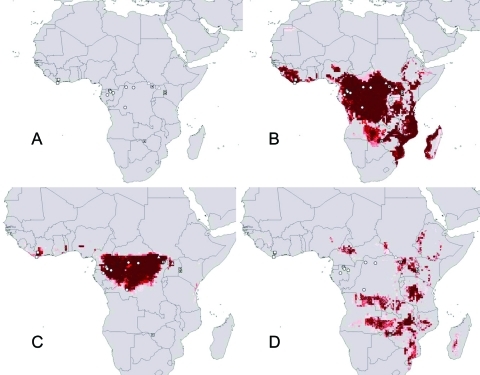
Summary of known and predicted geography of filoviruses in Africa. (A) Known occurrence points of filovirus hemorrhagic fevers (HFs) identified by virus species. (B) Geographic projection of ecologic niche model based on all known filovirus disease occurrences in Africa. (C) Geographic projection of ecologic niche model based on all known Ebola HF occurrences (i.e., eliminating Marburg HF occurrences). (D) Geographic projection of ecologic niche model based on all known occurrences of Marburg HF (i.e., eliminating Ebola HF occurrences). Darker shades of red represent increasing confidence in prediction of potential presence. Open squares, Ebola Ivory Coast; circles, Ebola Zaire; triangles, Ebola Sudan; dotted squares, Marburg HF occurrences.

 The geographic distribution of filovirus disease spreads generally across the humid Afrotropics (Figure 1A). Outlier occurrences lie at the eastern extreme of the distribution, consisting of occurrences associated with Ebola Sudan and Marburg viruses. Preliminary analyses of these geographic distributions, based on random subsets of the few data points available, indicated high statistical significance to model predictions: predictions of the geographic distribution of filovirus HFs correctly included random independent subsets much better than random model expectations (all p < 10^-7^). Although subsequent modeling was done without subsetting to maximize occurrence data, these preliminary results nonetheless indicated excellent predictivity of our distributional hypoptheses.

 Modeling the distribution of *Filoviridae* in general (all points in Figure 1A) produced a broad potential distribution across the Afrotropics, including areas from which filovirus HF occurrences have not been reported (Tanzania, Mozambique, Madagascar; Figure 1B). Predicted distributions of the two major *Filoviridae* clades—Ebola and Marburg viruses—showed different geographic patterns. When just the three African Ebola virus species were analyzed together, areas of overprediction in eastern Africa disappeared, and predicted distributional areas included only areas surrounding known occurrence points, except for a few small disjunct areas in West Africa (Figure 1C). The predicted distribution did not include all of the Afrotropics—coastal central Africa and most of West Africa appeared not to be included, although these models are based on very small samples of occurrences.

 When we analyzed the relatively few Marburg HF occurrences for which distributional data exist (n = 4 occurrences), a complementary distributional area was predicted (Figure 1D). Marburg HF was predicted to be absent in the humid Afrotropics, rather appearing focused in drier areas in eastern and south-central Africa. In contrast to Ebola HF, Marburg virus appears to have the potential to occur in areas from which filovirus disease has not yet been described.

**Figure 2:**
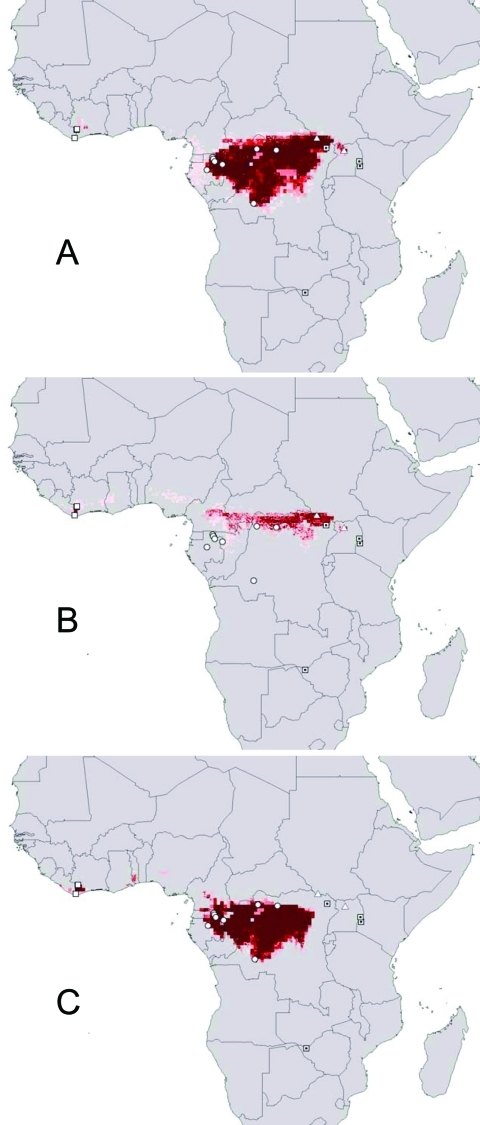
Geographic projection of ecologic niche models in which two Ebola virus species were modeled and used to predict the distributional area of the third. (A) Ebola Zaire and Ebola Sudan predicting (Ebola Ivory Coast omitted; note that distributional area is predicted in Ivory Coast). (B) Ebola Sudan and Ebola Ivory Coast predicting (Ebola Zaire omitted). (C) Ebola Zaire and Ebola Ivory Coast predicting (Ebola Sudan omitted). Darker shades of red represent increasing confidence in prediction of potential presence. Open squares, Ebola Ivory Coast; circles, Ebola Zaire; triangles, Ebola Sudan; dotted squares, Marburg hemorrhagic fever occurrences.

 Sequential omission of Ebola virus species from analyses provided a view of ecologic similarity of species (*45*): if omission of a particular species causes little overall change, then its ecologic characteristics are not distinct from those of the remaining species. Omission of Ebola Ivory Coast had little effect on the prediction (Figure 2A; note predicted area in Ivory Coast); similarly, predictions omitting Ebola Zaire included at least part of the distribution of Ebola Zaire (southern portion omitted; Figure 2B). Eliminating Ebola Sudan, however, yielded a prediction completely excluding the distribution of Ebola Sudan (Figure 2C), which suggests that Ebola Sudan occurs under a distinct ecologic regime.

**Figure 3:**
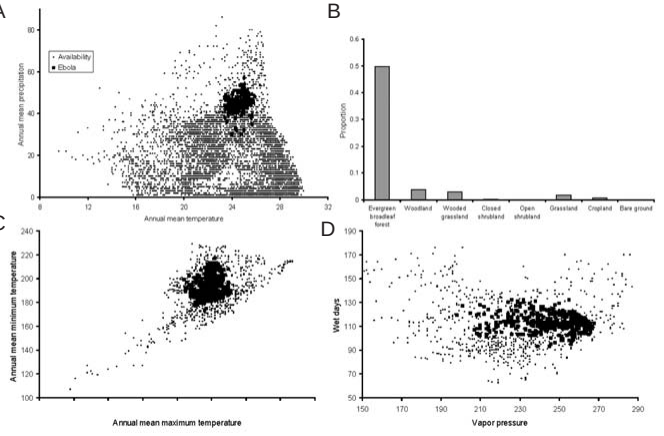
Ecologic distribution of predicted potential distributional areas for Ebola hemorrhagic fever (HF) occurrences, visualized in a few dimensions of climate. (A) Large-scale view (all of Africa), in which the basic concentration of Ebola HF occurrences in hot, wet climates is shown. (B) Distribution by land use/land-cover type, summarized as the proportion of overall area of land-cover types that is predicted to be present at the highest confidence level. (C,D) Regional scale (distributional area predicted by all 20 best-subsets models for Ebola HF buffered by 200 km in all directions) view of the ecology of occurrences of Ebola HF, visualized in dimensions of annual mean minimum temperature, annual mean maximum temperature, wet days, and vapor pressure.

 Inspection of niche models of Ebola HF occurrences (Marburg HF excluded) in ecologic space (Figure 3) provided insight into their ecologic distribution. Predicted Ebola HF occurrences were concentrated in regions presenting high precipitation combined with moderate-to-high temperatures (Figure 3A), coinciding with the ecologic distribution of evergreen broadleaf forest, although in specific cases that forest may be highly disturbed. In fact, >50% of African evergreen broadleaf forest is predicted to be within the niche of Ebola HF; no other land-cover type exceeded 5% within the Ebola HF niche (Figure 3B). In other dimensions, Ebola HF occurrences were distributed centrically in African environments and did not include extremes (Figure 3C–D).

**Figure 4:**
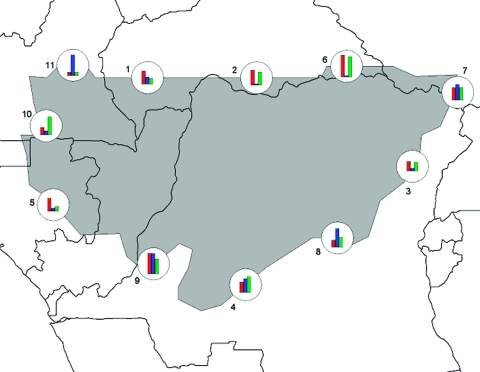
Preliminary exploration of patterns of ecologic variation along the modeled distributional limits (highest confidence level) for Ebola viruses in central Africa. The histograms represent relative values of Mann-Whitney U-tests for inside versus outside the prediction area for temperature (red bars), precipitation (blue bars), and elevation (green bars).

 Distributional limits are complex results of multiple causal agents. A species is seldom limited on all sides by a single factor; rather, distributional limits are the combined result of many such factors. Inspection of the ecologic dimensions coincident with modeled geographic limits of Ebola HF occurrences (Figure 4) showed some of this complexity. At points around the distributional limit of Ebola HF distributional areas in central Africa, precipitation dominates the range limit at point 11, but temperature and elevation dominate at points 2, 3, and 6. Moreover, gradients are steeper in some areas than others (e.g., point 6 vs. 3). This preliminary analysis thus illustrates the complex relationships between ecologic dimensions and distributional limits.

**Figure 5:**
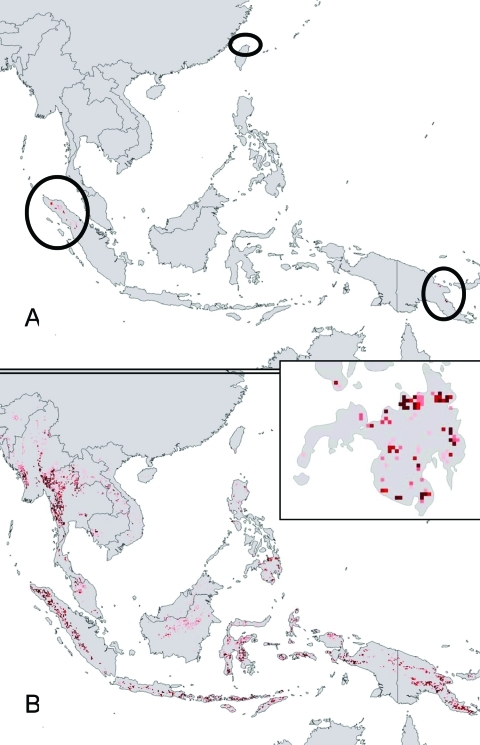
Projection of filovirus ecologic niche models onto southeastern Asia and the Philippines to assess the degree to which possible Philippine distributional areas are predictable on the basis of the ecologic characteristics of African filovirus hemorrhagic fever (HF) occurrences. (A) Projection of model for Marburg HF occurrences (Figure 1D) to southeastern Asia. (B) Projection of model for all filovirus disease occurrences (Figure 1B) to southeastern Asia (the projection of models for Ebola HF occurrences is identical to this map). Inset: detail of projection to the island of Mindanao, in the Philippines. Darker shades of red represent increasing confidence in prediction of potential presence.

 Given the mysterious origin of Ebola Reston virus (Ebola HF among macaques in a breeding facility on Luzon, Philippines) (*9*,*13*), a key question regarding Ebola HF distribution and ecology is whether similar ecologic conditions exist in Southeast Asia (e.g., Philippines). Projecting ecologic niche models for Marburg HF in Africa onto Asian environments identified few “appropriate” areas: only a few scattered areas in Papua New Guinea and Indonesia (Figure 5A). Projection of Ebola HF models, however, identified broader potential distributional areas in Southeast Asia (Figure 5B), including the lowlands of Mindanao (Figure 5, inset), a finding that suggests that similar ecologic conditions exist in the Philippines.

## Discussion

### Ecology and Geography of Filovirus Occurrences

The ecologic niche characteristics reconstructed for filovirus species disease outbreaks coincided closely with phylogenetic patterns in the group (*1*,*48*). That is, disease sites for Ebola Ivory Coast and Ebola Zaire coincided ecologically, and these viruses are phylogenetically sister taxa. Ebola Sudan is genetically and ecologically most distinct among Ebola virus species, and (with Ebola Reston) forms the sister clade to Ebola Ivory Coast + Ebola Zaire. Correspondence between phylogenetic and ecologic patterns suggests that ecologic distributions of Ebola Sudan and Ebola Reston may prove similar; hence, the ecologic characteristics of Ebola Sudan may provide clues about the origin of Ebola Reston.

Marburg HF occurrence sites are quite distinct, with minimal overlap with Ebola HF ecologic distributions, coinciding with Marburg virus’ distant position in the phylogeny of the *Filoviridae*. This pattern suggests that Marburg virus and the Ebola viruses may have host species with markedly different ecologic requirements.

### Ebola Reston

The geographic origin of Ebola Reston virus has been subject of controversy (*9*,*49*). Although the Ebola virus-infected monkeys initially documented in Reston, Virginia, originated in the Philippines, whether Ebola Reston occurs naturally in the Philippines has been debated. Nevertheless, the virus is distinct, and its geographic distribution is otherwise unknown. Given the phylogeny-ecology correspondence documented above, the ecology of Ebola Sudan may prove key in predicting the distribution of Ebola Reston, but the scanty occurrence data make species-specific models difficult. Our results are relevant in that ecologic conditions under which Ebola HF occurs in Africa are also found in the Philippines.

In previous analyses of animals, the conservative nature of ecologic niches has been documented to lead to prediction into regions inhabited by congener species (*45*). To the extent that host-parasite codistribution and cospeciation may be involved in the virus-reservoir relationships of filoviruses, prediction of potential distributional areas in the Philippines may reflect conservative niche evolution in the host taxon. Of course, because of historical effects (e.g., limited dispersal) on species’ distributions, potential distributional areas are often predicted in areas not inhabited (*44*), so this evidence is not definitive.

### Limitations of the Approach

Limitations of our approach should be recognized. First, small sample sizes become critical. Although predictive models can be developed with relatively small samples of occurrence points (*39*), sample sizes for filovirus HF disease outbreaks are so minimal that single data points can change overall results. Examples of this sensitivity include the Zimbabwe Marburg HF disease outbreak and the Booue, Gabon, Ebola Zaire HF outbreak; inclusion of these points causes geographic predictions to be expanded considerably.

Other limitations center on the ecologic dimensions in which the niche is modeled. If additional dimensions exist that limit species’ distributions (and they certainly do) , GARP predictions will be overly large. Jackknife manipulations (systematic omission of ecologic dimensions to assess sensitivity to coverage density) can, to some degree, help in assessing sensitivity to coverage completeness *(42)*, but dimensions more important than the set actually used may exist. Particularly relevant is climate variability—extreme events such as droughts and heavy rainfall may prove particularly relevant to filovirus transmission but are not included herein; such more complex models are under development (A.T. Peterson et al., unpub. data). Spurious associations between occurrence points and ecologic dimensions, though usually detected through independent test datasets, can limit distributional predictions overmuch.

### Natural Reservoirs for Filoviruses

Detailed understanding of the geography and ecology of filovirus HF outbreaks represents an underexplored avenue of investigation regarding natural transmission cycles of filoviruses. We assembled available information regarding filovirus HF outbreaks and used various analytical tools to arrive at a detailed understanding of geography and ecology of filovirus disease occurrences. Consequently, we can now assemble criteria by which potential reservoir taxa might be judged. If one assumes a fair degree of host specificity in this host-parasite system, patterns of codistribution and cophylogeny can be expected. Hence, criteria include the following: 1) African Ebola virus reservoirs would be distributed principally in evergreen broadleaf forest; 2) the main focus of the geographic distribution of the reservoir(s) would be in the Congo Basin; 3) a disjunct (allopatric) distributional area would be present in West Africa; 4) a related taxon in eastern Africa would range in more arid habitats; 5) the reservoir would belong to a clade more broadly distributed across Africa and Southeast Asia.

 Assessment of potential reservoir taxa by using these criteria has begun (A.T. Peterson et al., unpub. data), with the idea of eventually testing hypotheses of host associations through ecologic niche comparison methods (*22*). The goal, to be explored in future publications, is to develop reduced lists of taxa of highest priority for virus survey.

## Footnotes

1Although filovirus taxonomy undergoes frequent revision, we follow nomenclature most recently established by the International Committee of Taxonomy of Viruses in 2002. Filoviruses consist of two genera. The genus *Marburgvirus* contains one species, *Lake Victoria* *marburgvirus*, with several recognized strains, and *Ebolavirus* contains four species: *Ivory Coast ebolavirus*, *Reston ebolavirus*, *Sudan ebolavirus*, and *Zaire ebolavirus*. For simplicity, we refer to the viruses using the unitalicized vernacular (e.g., Ebola Zaire). We use “Ebola viruses” to refer in general to members of *Ebolavirus* and “Marburg viruses” to refer in general to members of *Marburgvirus*. The diseases caused by filoviruses are termed Ebola hemorrhagic fever (HF; diseases caused by Ebola viruses) and Marburg HF (diseases caused by Marburg viruses).
